# Novel lymph node ratio predicts prognosis of colorectal cancer patients after radical surgery when tumor deposits are counted as positive lymph nodes: a retrospective multicenter study

**DOI:** 10.18632/oncotarget.12076

**Published:** 2016-09-16

**Authors:** Jin Yang, Shasha Xing, Jun Li, Shengke Yang, Junjie Hu, Hao Liu, Feng Du, Jie Yin, Sai Liu, Ci Li, Jiatian Yuan, Bo Lv

**Affiliations:** ^1^ Central Laboratory, Affiliated Hospital/Clinical Medical College of Chengdu University, Chengdu, China; ^2^ General Surgery Department, Affiliated Hospital/Clinical Medical College of Chengdu University, Chengdu, China; ^3^ Department of General Surgery, Zhongshan Hospital, Fudan University Colorectal Cancer Research Center, Shanghai, China; ^4^ General Surgery Department, Sichuan Cancer Hospital, Chengdu, China; ^5^ Gastrointestinal Tumor Surgery Department, Hubei Cancer Hospital, Wuhan, China; ^6^ General Surgery Department, 2nd Affiliated Hospital of Jilin University, Changchun, China; ^7^ Internal Medicine-Oncology, Cancer Institute, Peking Union Medical College and Chinese Academy of Medical Sciences, Beijing, China; ^8^ General Surgery Department, Xuzhou Central Hospital, Xuzhou, China; ^9^ Surgical Department of Gastrointestinal Diseases, Youan Hospital of Capital Medical University, Beijing, China; ^10^ Department of Pathology, Clinical Medical College of Chengdu University, Chengdu, China

**Keywords:** lymph node ratio, tumor deposits, colorectal cancer, prognosis

## Abstract

The lymph node ratio (LNR), defined as the relation of tumor-infiltrated to resected lymph nodes, has been identified as an independent prognostic factor for colorectal cancer (CRC) after radical surgery. Recently, new guidelines propose counting tumor deposits (TDs) as positive lymph nodes (pLNs). The aim of this study was to investigate whether a novel LNR (nLNR) that considers TDs as pLNs can be used to accurately predict the long-term outcome of CRC patients. In this multicenter retrospective study, clinicopathological and outcome data from 2,051 stage III CRC patients who underwent R0 resection were collected between January 2004 and December 2011. Disease-free survival (DFS) and overall survival (OS) according to the nLNR category were analyzed using Kaplan-Meier survival curves. Univariate and multivariate analyses were performed to determine significant prognostic factors, and ROC curves were computed to measure the predictive capacity of the nLNR category. The 5-year DFS rates of nLNR1-4 were 68.3%, 48.4%, 33.3% and 16.5%, respectively (*P*<0.0001), and the 5-year OS rate of nLNR1-4 were 71.8%, 60.1%, 42.7% and 21.8%, respectively (*P*<0.0001). The area of under curve (AUC) of the nLNR was 0.686 (95% CI 0.663-0.710) and 0.672 (95% CI 0.648-0.697) for predicting DFS and OS. Our results demonstrate that the nLNR predicted long-term outcomes better than the LNR, npN and pN, using the cutoff points 0.250, 0.500 and 0.750.

## INTRODUCTION

The presence of tumor deposits (TDs) is a prognostic indicator for colorectal cancer (CRC) [1–4]. The TNM staging system by the American Joint Committee on Cancer (AJCC) issues definitions of what should be considered as positive lymph nodes (pLNs) and TDs [5–7]. The TNM5 classification [5] was the first to evaluate TDs and proposed the 3-mm rule. Later, the TNM6 [6] defined TDs as pLNs when they had the form and smooth contour of LNs while irregular TDs remained in the T category. In 2010, a new pN1c category was defined in TNM7 [7], which considered that T1 and T2 lesions with tumor deposits but lacking regional positive lymph node(s) should be classified as pN1c. This rapidly changing classification criteria impacts the selection of strategies to treat patients, such as postoperative chemoradiotherapy. Recently discussions to simplify the TNM staging system address whether TDs should be considered pLNs [1, 8]. A large-scale study by Li J *et al* reported that the counting TDs as pLNs in the new pN (npN) category is potentially superior to the classification in the original pN category, in terms of evaluating long-term outcomes for CRC patients [8].

The “lymph node ratio” (LNR) has been used as a predictive factor of the long-term survival status of CRC patients after radical surgery [9, 10]. Here, we conducted a large-scale, multicenter study, analyzing data from 2,051 stage-III CRC patients who received initial radical surgery, in order to investigate whether nLNR (relation of number of pLNs plus TDs to all nodes in resected samples) can accurately predict the long-term outcomes such as 5-year disease-free survival (DFS) and overall survival (OS).

## RESULTS

### Harvested LNs status and TDs

We harvested a total of 32,505 nodes including 30,707 LNs and 1,798 TDs, in which there were 7,771 pLNs. The number of pLNs plus TDs added to a total of 9,569 positive nodes. The mean number of retrieved LNs was 15.0. The LNR (pLNs to total LNs, 7,771/30,707) was 0.253, while the nLNR (positive nodes to all nodes, 9,569/32,505) was 0.294. We found that 35.0% (717/2,051) of patients had TDs, and there were 39.3% (282/717) of patients with TDs but without pLNs.

### Patient characteristics and association of nLNR category with clinicopathologic factors

The median age was 58.0 ± 12.3 years (range 14-84), and the ratio of male to female was 1.26: 1 (1,144/907). In this study, only patients with adenocarcinoma including tubular (91.9%), mucinous (6.1%) or ring cell (2.0%) tumors were included. Patient clinicopathologic features are listed in Table 1. The rates of nLNR categories 1-4 were 53.9%, 26.4%, 11.6%, and 8.1%, respectively. nLNR categories were associated with tumor location (colon or rectum), pT category (7th), pN (7th) or npN category, venous/lymphatic invasion, differentiation grade, pathological category, and histological type (all *P*<0.05). The distributions of nLNR subgroups were similar with respect to gender, age, tumor size, preoperative carcinoembryonic antigen (CEA) levels, or adjuvant chemoradiotherapy (*P*>0.05, respectively).

**Table 1:** Association of nLNR category with clinical and pathological characteristics

Variable	All Patients		nLNR Category								X^2^	P
	No.	%	nLNR1	%	nLNR2	%	nLNR3	%	nLNR4	%		
**All patients**	2051	100.0%	1105	53.9%	542	26.4%	238	11.6%	166	8.1%		
**Gender**												
** Male**	1144	55.8%	630	57.0%	301	55.5%	125	52.5%	88	53.0%	2.236	0.525
** Female**	907	44.2%	475	43.0%	241	44.5%	113	47.5%	78	47.0%		
**Age, year**												
** ≤60**	1187	57.9%	630	57.0%	296	54.6%	143	60.1%	118	71.1%	3.622	0.057
** >60**	864	42.1%	475	43.0%	246	45.4%	95	39.9%	48	28.9%		
**Tumor location**												
** Colon**	635	31.0%	393	35.6%	154	28.4%	52	21.8%	36	21.7%	18.865	<0.0001
** Rectum**	1416	69.0%	712	64.4%	388	71.6%	186	78.2%	130	78.3%		
**Tumor size, diameter**												
** ≤5.0 cm**	1440	70.2%	748	67.7%	418	77.1%	167	70.2%	107	64.5%	5.693	0.128
** >5.0 cm**	611	29.8%	357	32.3%	124	22.9%	71	29.8%	59	35.5%		
**Preoperative CEA levels**												
** <5.0 ng/ml**	1102	53.7%	607	54.9%	297	54.8%	118	49.6%	80	48.2%	9.169	0.164
** ≥5.0 ng/ml**	704	34.3%	378	34.2%	169	31.2%	92	38.7%	65	39.2%		
** Unknown**	245	11.9%	120	10.9%	76	14.0%	28	11.8%	21	12.7%		
**pT category (7th)**												
** pT2**	128	6.2%	81	7.3%	36	6.6%	7	2.9%	4	2.4%	18.107	0.006
** pT3**	642	31.3%	350	31.7%	181	33.4%	69	29.0%	42	25.3%		
** pT4**	1281	62.5%	674	61.0%	325	60.0%	162	68.1%	120	72.3%		
**pN category (7th)**												
** pN1a**	533	26.0%	476	43.1%	51	9.4%	4	1.7%	2	1.2%	117.1	<0.0001
** pN1b**	539	26.3%	342	31.0%	156	28.8%	37	15.5%	4	2.4%		
** pN1c**	282	13.7%	197	17.8%	79	14.6%	4	1.7%	2	1.2%		
** pN2a**	374	18.2%	80	7.2%	185	34.1%	88	37.0%	21	12.7%		
** pN2b**	323	15.7%	10	0.9%	71	13.1%	105	44.1%	137	82.5%		
**npN category**												
** pN1a**	526	25.6%	508	46.0%	18	3.3%	0	0.0%	0	0.0%	136.21	<0.0001
** pN1b**	629	30.7%	453	41.0%	156	28.8%	16	6.7%	4	2.4%		
** pN2a**	495	24.1%	134	12.1%	258	47.6%	82	34.5%	21	12.7%		
** pN2b**	401	19.6%	10	0.9%	110	20.3%	140	58.8%	141	84.9%		
**Venous/lymphatic invasion**												
** Yes**	230	11.2%	75	6.8%	66	12.2%	38	16.0%	51	30.7%	75.987	<0.0001
** No**	1821	88.8%	1030	93.2%	476	87.8%	200	84.0%	115	69.3%		
**Differentiation grade**												
** Well**	208	10.1%	126	11.4%	62	11.4%	14	5.9%	6	3.6%	215.17	<0.0001
** Moderately**	1507	73.5%	877	79.4%	401	74.0%	80	33.6%	80	48.2%		
** Poorly**	336	16.4%	102	9.2%	79	14.6%	75	31.5%	80	48.2%		
**Pathological category**												
** Tubular adenocarcinoma**	1885	91.9%	1042	94.3%	502	92.6%	220	92.4%	121	72.9%	83.956	<0.0001
** Mucinous adenocarcinoma**	125	6.1%	54	4.9%	34	6.3%	6	2.5%	31	18.7%		
** Ring cell cancer**	41	2.0%	9	0.8%	6	1.1%	12	5.0%	14	8.4%		
**Histological type**												
** Protrude**	1250	60.9%	684	61.9%	345	63.7%	144	60.5%	77	46.4%	68.773	<0.0001
** Ulcer**	638	31.1%	336	30.4%	180	33.2%	75	31.5%	47	28.3%		
** Infiltrative**	163	7.9%	85	7.7%	17	3.1%	19	8.0%	42	25.3%		
**Adjuvant therapy**												
** Yes**	1827	89.1%	991	89.7%	486	89.7%	220	92.4%	130	78.3%	10.261	0.097
** No**	224	10.9%	114	10.3%	56	10.3%	18	7.6%	36	21.7%		

### LNR versus nLNR as a prognostic for DFS and OS

During a 61-months (median; range 2-136) follow-up visit, a total of 838 patients (43.1%) were detected with failure including 11.5% of patients (235/2,051) with LR, 34.2% (701/2,051) with DM, 4.8% (98/2,051) with both LR and DM. For all 2,051 patients, the rates of 5-year DFS and OS were 55.1% and 62.0%, respectively. Table 2 lists the association of clinical and pathologic factors with long-term outcomes. The 5-year DFS rates of nLNR1-4 were 68.3%, 48.4%, 33.3% and 16.5%, respectively (*P*<0.0001). By contrast, the 5-year rates of DFS for LNR1-4 were 66.7%, 41.0%, 37.3% and 16.6%, respectively (*P*<0.0001). The 5-year OS for nLNR1-4 were 71.8%, 60.1%, 42.7% and 21.8%, respectively (*P*<0.0001). Compared to the nLNR category, the 5-year OS for LNR1-4 were 71.7%, 53.4%, 40.6% and 20.3%, respectively (*P*<0.0001).

**Table 2:** Impact of clinical and pathological characteristics on 5-year DFS and OS

Variable	All patients	All Failure (LR and/or DM)		5-Years DFS Rate	X^2^	*P*	5-Year OS Rate	X^2^	*P*
		No. cases	%						
**All patients**	2051	883	43.1%	55.1%			62.0%		
**Gender**									
** Male**	1144	502	43.9%	54.1%	0.065	0.800	62.0%	2.963	0.085
** Female**	907	381	42.0%	56.2%			60.2%		
**Age, year**									
** ≤60**	1187	497	41.9%	57.1%	0.078	0.005	64.1%	12.258	<0.0001
** >60**	864	386	44.7%	52.1%			57.1%		
**Tumor location**									
** Colon**	635	270	42.5%	56.7%	0.100	0.751	62.9%	0.609	0.435
** Rectum**	1416	613	43.3%	54.2%			60.5%		
**Tumor size, diameter**									
** ≤5.0 cm**	1440	608	42.2%	56.3%	8.186	0.004	66.8%	19.960	<0.0001
** >5.0 cm**	611	275	45.0%	52.2%			56.0%		
**Preoperative CEA levels**									
** <5.0 ng/ml**	1102	404	36.7%	62.4%	29.562	<0.0001	68.9%	61.925	<0.0001
** ≥5.0 ng/ml**	704	363	51.6%	45.3%			51.2%		
**Unknown**	245	116	47.3%	49.5%			53.9%		
**pT category (7th)**									
** pT2**	128	26	20.3%	78.6%	80.647	<0.0001	82.8%	61.982	<0.0001
** pT3**	642	226	35.2%	63.5%			68.5%		
** pT4**	1281	631	49.3%	48.4%			55.3%		
**pN category (7th)**									
** pN1a**	533	149	28.0%	71.5%	210.812	<0.0001	74.3%	241.467	<0.0001
** pN1b**	539	215	39.9%	57.8%			63.7%		
** pN1c**	282	87	30.9%	69.9%			73.9%		
** pN2a**	374	207	55.3%	39.8%			49.9%		
** pN2b**	323	225	69.7%	25.7%			32.9%		
**npN category**									
** pN1a**	526	141	26.8%	72.4%	243.677	<0.0001	77.2%	252.334	<0.0001
** pN1b**	629	207	32.9%	65.6%			68.4%		
** pN2a**	495	252	50.9%	46.0%			57.2%		
** pN2b**	401	283	70.6%	26.0%			35.9%		
**Venous/lymphatic invasion**									
** Yes**	230	151	65.7%	28.1%	82.275	<0.0001	34.7%	84.399	<0.0001
** No**	1821	732	40.2%	58.3%			64.3%		
**Differentiation grade**									
** Well**	208	56	26.9%	71.8%	77.730	<0.0001	75.4%	86.138	<0.0001
** Moderately**	1507	627	41.6%	56.6%			63.1%		
** Poorly**	336	200	59.5%	37.4%			42.6%		
**Pathological category**									
** Tubular adenocarcinoma**	1885	799	42.4%	55.7%	12.542	0.002	62.5%	32.689	<0.0001
** Mucinous adenocarcinoma**	125	58	46.4%	55.2%			50.4%		
** Ring cell cancer**	41	26	63.4%	34.7%			35.6%		
**Histological type**									
** Protrude**	1250	496	39.7%	58.9%	32.074	<0.0001	63.8%	20.552	<0.0001
** Ulcer**	638	298	46.7%	50.9%			58.7%		
** Infiltrative**	163	89	54.6%	41.0%			50.3%		
**Adjuvant therapy**									
** Yes**	1827	771	42.2%	55.8%	11.710	0.001	63.9%	30.826	<0.0001
** No**	224	112	50.0%	50.4%			51.7%		
**LNR category**									
** LNR1**	1270	412	32.4%	66.7%	222.124	<0.0001	71.7%	245.392	<0.0001
** LNR2**	442	245	55.4%	41.0%			53.4%		
** LNR3**	186	107	57.5%	37.3%			40.6%		
** LNR4**	153	119	77.8%	16.6%			20.3%		
**nLNR category**									
** nLNR1**	1105	339	30.7%	68.3%	300.214	<0.0001	71.8%	261.948	<0.0001
** nLNR2**	542	266	49.1%	48.4%			60.1%		
** nLNR3**	238	150	63.0%	33.3%			42.7%		
** nLNR4**	166	128	77.1%	16.5%			21.8%		

**Figure 1:**
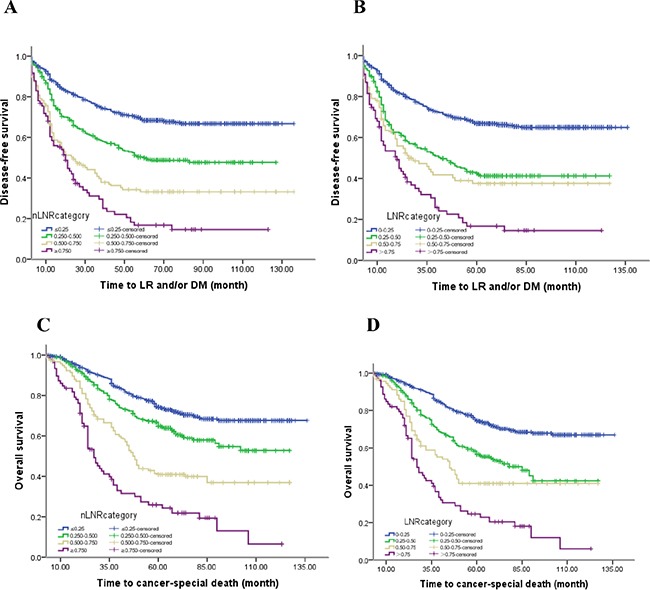
The DFS and OS curves for nLNR and LNR categories The 5-year DFS rates for **A.** the nLNR category (68.3%, 48.4%, 33.3% and 16.5%, *P*<0.0001; all statistically different, *P*<0.005), and **B.** the LNR category (66.7%, 41.0%, 37.3% and 16.6%, *P*<0.0001; all statistically different, *P*<0.001, except LNR 2 versus LNR 3; X^2^=1.989, *P*=0.158), The 5-year OS rates of **C.** the nLNR category (71.8%, 60.1%, 42.7% and 21.8%, *P*<0.0001; all statistically different, *P*<0.0001), and **D.** the LNR category (71.7%, 53.4%, 40.6% and 20.3%, *P*<0.0001; all statistically different, *P*<0.002).

The results of univariate analysis indicated that these thirteen clinical or pathologic factors including age, tumor size, preoperative CEA levels, pT or pN category, npN category, venous/lymphatic invasion, differentiation grade, pathological category, histological type, adjuvant chemoradiotherapy, LNR and nLNR categories, were all correlated with DFS and OS (all *P*<0.05). On the other hand, gender and tumor location could not predict the long-term outcomes of CRC patients (all *P*>0.05). Figure 1 shows the DFS and OS curves for both LNR and nLNR categories.

**Figure 2:**
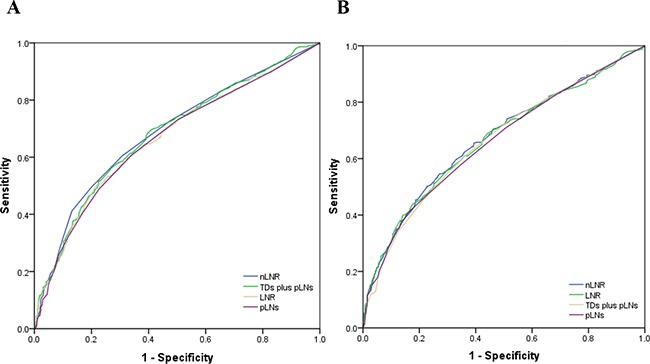
The ROC curves of nLNR, npLNs (TDs plus pLNs), LNR, and pLNs for predicting DFS and OS in all patients **A.** The AUC of nLNR, npLNs, LNR, and pLNs for predicting DFS were: 0.686 (95% CI 0.663-0.710), 0.681 (95% CI 0.657-0.704), 0.668 (95% CI 0.644-0.691), and 0.664 (95% CI 0.640-0.688), respectively (all *P*<0.0001). **B.** The AUC of nLNR, LNR, npLNs, and pLNs for predicting OS were: 0.672 (95% CI 0.648-0.697), 0.667 (95% CI 0.643-0.692), 0.663 (95% CI 0.638-0.688), and 0.659 (95% CI 0.635-0.684), respectively (all *P*<0.0001).

**Figure 3:**
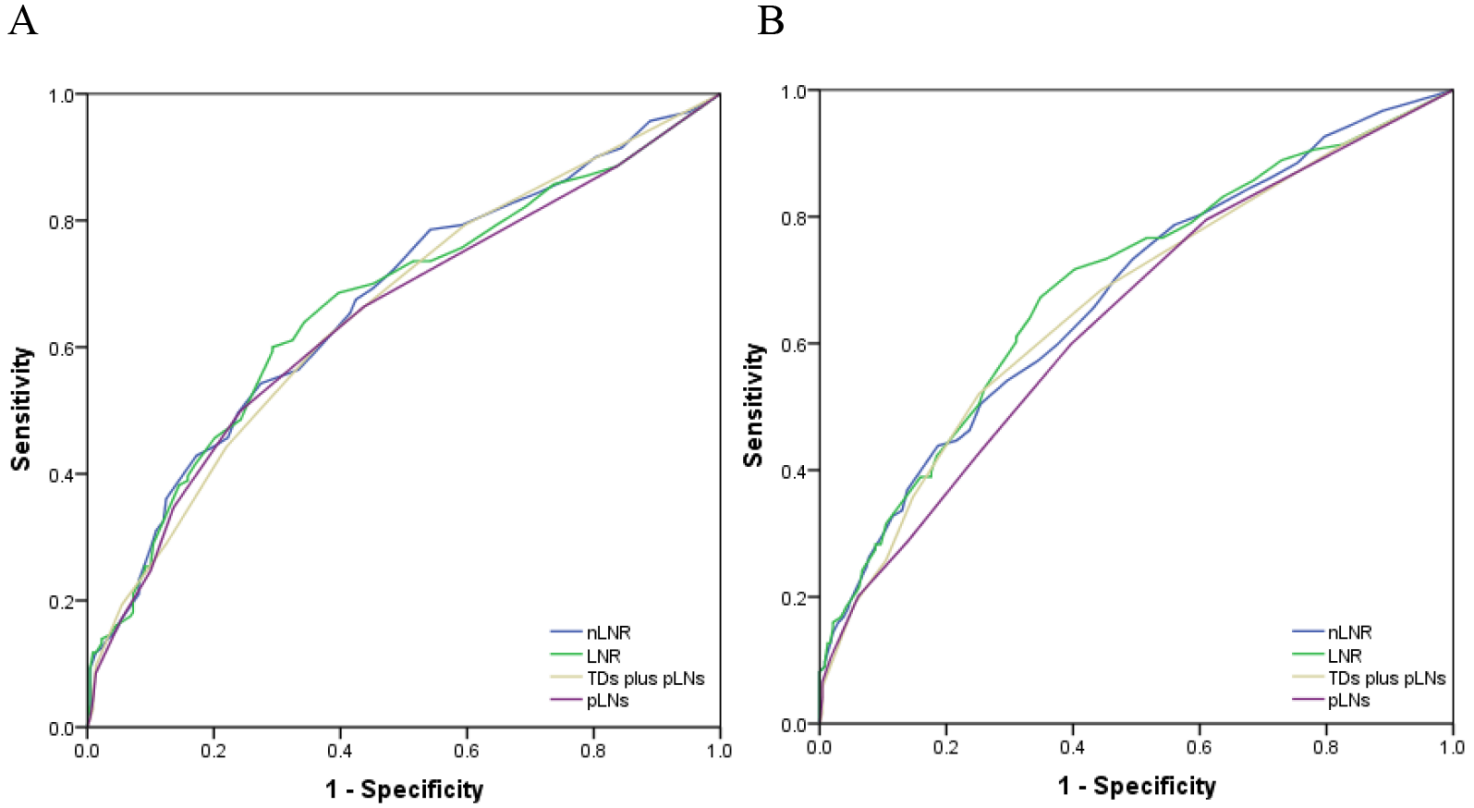
The ROC curves of nLNR, npLNs (TDs plus pLNs), LNR, and pLNs for predicting DFS and OS in patients with less than 12 nodes **A.** The AUC of nLNR, npLNs, LNR, and pLNs for predicting DFS were: 0.670 (95% CI 0.629-0.711), 0.666 (95% CI 0.625-0.708), 0.658 (95% CI 0.616-0.699), and 0.648 (95% CI 0.606-0.690), respectively (all *P*<0.0001). **B.** The AUC of nLNR, LNR, npLNs, and pLNs for predicting OS were: 0.675 (95% CI 0.634-0.717), 0.689 (95% CI 0.647-0.730), 0.664 (95% CI 0.621-0.707), and 0.640 (95% CI 0.597-0.683), respectively (all *P*<0.0001).

In a previous study, we indicated that the npN category was superior to the pN category as a predictor of prognosis. In addition to the high correlation with each other, only the npN category was considered in multivariate models. Given that nLNR and LNR were highly correlated, multivariate analyses for all patients assessed both variables separately to avoid potential bias (Table 3 and 4). Both the nLNR and LNR categories were determined to be independent prognostic factors for DFS (HR 1.497, 95% CI 1.306 to 1.576, *P* < 0.0001; HR 1.411, 95% CI 1.294 to 1.537, *P*=0.001) and OS (HR 1.425, 95% CI 1.291 to 1.583; HR 1.418, 95% CI 1.288 to 1.561, both *P*< 0.0001). In order to identify which variable was superior in predicting DFS and OS, the area under the curve (AUC) was calculated for nLNR and LNR ROC curves. nLNR was found to be superior to LNR (AUC = 0.686, 95% CI 0.663-0.710 vs. 0.668, 95% CI 0.644-0.691) in predicting DFS. Similarly, nLNR was a more accurate indicator than LNR to assess OS (AUC = 0.672, 95% CI 0.648-0.697 vs. 0.667, 95% CI 0.643-0.692) (Figure 2). For those patients with less than 12 nodes (TDs plus pLNs), nLNR was also superior to LNR in assessing DFS (AUC = 0.670, 95%CI 0.629-0.711 vs. 0.666, 95% CI 0.625-0.708) and OS (AUC = 0.675, 95% CI 0.634-0.717 vs. 0.689, 95% CI 0.647-0.730) (Figure 3).

**Table 3:** Multivariate analysis for 5-year DFS and OS when nLNR enrolled

Variables	5-Year DFS			5-Year OS		
	HR	95.0% CI	*P*	HR	95.0% CI	*P*
**Age**	1.139	(1.139 to 1.307)	0.063	1.306	(1.127 to 1.514)	0.000
**Tumor size**	0.975	(0.975 to 1.135)	0.747	1.042	(0.887 to 1.225)	0.615
**Preoperative CEA levels**	0.857	(0.769 to 0.956)	0.006	0.890	(0.792 to 1.000)	0.051
**pT category (7th)**	1.287	(1.198 to 1.396)	0.000	1.249	(1.196 to 1.463)	0.000
**npN category**	1.164	(1.065 to 1.271)	0.000	1.186	(0.571 to 0.857)	0.000
**Venous/lymphatic invasion**	0.671	(0.557 to 0.810)	0.000	0.700	(0.571 to 0.857)	0.001
**Differentiation grade**	1.194	(1.037 to 1.374)	0.014	1.217	(1.047 to 1.414)	0.010
**Pathological category**	0.937	(0.790 to 1.110)	0.454	1.094	(0.920 to 1.301)	0.308
**Histological type**	1.099	(0.991 to 1.218)	0.072	1.058	(0.946 to 1.184)	0.323
**Adjuvant therapy**	1.324	(1.136 to 1.544)	0.000	1.369	(1.122 to 1.560)	0.000
**LNR category**	1.411	(1.294 to 1.537)	0.001	1.418	(1.288 to 1.561)	0.000

**Table 4:** Multivariate analysis for 5-year DFS and OS when LNR enrolled

Variables	5-Year DFS			5-Year OS		
	HR	95.0% CI	*P*	HR	95.0% CI	*P*
**Age**	1.133	(0.988 to 1.300)	0.074	1.296	(1.118 to 1.501)	0.001
**Tumor size**	0.980	(0.842 to 1.420)	0.790	1.049	(0.893 to 1.233)	0.560
**Preoperative CEA levels**	0.853	(0.853 to 0.952)	0.004	0.880	(0.783 to 0.990)	0.033
**pT category (7th)**	1.240	(1.176 to 1.504)	0.000	1.301	(1.256 to 1.491)	0.000
**npN category**	1.337	(1.216 to 1.469)	0.000	1.145	(1.035 to 1.267)	0.009
**Venous/lymphatic invasion**	0.673	(0.558 to 0.812)	0.000	0.684	(0.559 to 0.838)	0.000
**Differentiation grade**	1.198	(1.043 to 1.376)	0.011	1.265	(1.092 to 1.466)	0.002
**Pathological category**	0.934	(0.788 to 1.108)	0.435	1.116	(0.940 to 1.326)	0.211
**Histological type**	1.100	(0.788 to 1.108)	0.069	1.067	(0.953 to 1.194)	0.260
**Adjuvant therapy**	1.309	(1.112 to 1.527)	0.001	1.371	(1.129 to 1.566)	0.000
**nLNR category**	1.497	(1.306 to 1.576)	0.000	1.425	(1.291 to 1.583)	0.000

## DISCUSSION

Lymph node (LN) involvement has been widely identified as one of the most important predictor of poor prognosis, and the pN staging strategy has been established by the AJCC TNM staging system according to the number of positive lymph nodes (pLNs) [5–7]. Recently, however, there has been increasing evidence that the pN staging based on the number of pLNs alone may not predict the long-term outcomes of patients accurately [11, 12]. In addition, although tumor deposits (TDs) are taken into account in TNM7 for colorectal cancer (CRC), which defines a new pN1c category (TDs are considered as pN1c when pT1-2 lesions lack pLNs), it is difficult to discriminate TDs from all nodes that include LNs [8].

Considering the shortcomings of pN, efforts have been made to identify more reliable prognostic markers related to LN status, such as LNR. The LNR is the ratio of pLNs to all resected LNs. Indeed, the LNR might be a good predictor of long-term survival in CRC [9, 10, 13, 14]. Rosenberg *et al.* [9] analyzed the data from a total of 17,309 CRC patients who underwent resection with a 5.9-year follow-up, finding that the LNR category could be used as an independent prognostic factor. Nonetheless, since guidelines regarding the use of LNR are lacking, it is not used widely in clinical practice.

TDs predict poor prognosis in CRC. However, the pN staging strategy does not consider the impact of the number of TDs on prognosis; therefore, recent studies have investigated whether a TD could be counted as a pLN. Results from such studies prompted the definition of a new pN (npN) category to evaluate long-term outcomes for CRC, which takes into account the number of both pLNs and TDs [1]. We recently investigated the feasibility of using the npN category to predict prognosis, analyzing data from 4,021 CRC patients who received radical surgery [8]. In that study, we proposed that TDs counted as pLNs could simplify the pN category (without pN1c), and found that the npN category is superior to the pN category in predicting DFS and OS [8]. Using the npN category, we did not find a need to differentiate TDs from nodes when the LN structure disappears totally.

In the present study, due to “no need to distinguish TDs and pLNs” [8], we designed the nLNR category calculating the ratio between the number of TDs plus pLNs and the number of TDs plus all harvested LNs. Besides, unlike other studies using variable cutoff values to classify the nLNR, we chose the standard 0.250, 0.500 and 0.750 as cutoff values based on statistical analyses, thereby circumventing the poor reproducibility of the LNR category. We investigated the feasibility of using the nLNR category to predict prognosis in CRC. Our findings from univariate and multivariate analyses indicated that the nLNR category could be used as an independent prognostic factor of both DFS and OS for CRC after radical surgery, similar to the LNR category [9, 10, 13, 14]. However, our ROC curve analyses indicated that the nLNR was superior to the LNR, npN, and pN in assessing DFS and OS in CRC. Thus, it is feasible to use the nLNR category to predict the long-term prognosis of CRC patients with greater accuracy than LNR category.

For patients with preoperative chemotherapy, the total number of harvested nodes can be less than 12 [11, 15–17]. Yet, according to the TNM7 staging criteria, at least 12 nodes are necessary to accurately use the pN category. Therefore, the nLNR category may be helpful to select reasonable treatment strategies for patients with less than 12 nodes. Indeed, our results here showed that nLNR was better than LNR, npN or pN in predicting the long-term survival of patients with less than 12 nodes.

Although our large-scale study included multicenter databases, it suffered from several limitations such as its retrospective design and lack of more long-term follow-up visits by patients. Previous studies have reported that patients can suffer a continuous increase in local recurrence for up to 10 years [18]. A randomized controlled trial, with different therapeutic strategies with or without adjuvant chemotherapy after radical surgery, should be done to validate our results. Besides, Colon and rectal cancer have different staging and treatment algorithms, even though similar outcomes were identified in this series. Nonetheless, our results warrant further prospective studies as well as the use of the nLNR category with the cutoff values indicated to predict the long-term prognosis of CRC patients.

## PATIENTS AND METHODS

### Inclusion and exclusion criteria

We examined the records of 2,051 patients with stage III colorectal adenocarcinoma who received initial radical surgery at seven study sites in China between January 2004 and December 2011. We excluded patients that: 1) had distant metastasis detected pre- or peri-operatively; 2) suffered from colorectal cancer before; 3) had synchronous tumors; 4) received preoperative chemoradiotherapy; 5) had multiple adenocarcinomas; 6) died of surgical complications in the immediate postoperative period; 7) lacked complete pathological slides; 8) lost follow-up visit within 5 years.

### Surgical and adjuvant treatments

All patients received R0 resection without preoperative radiotherapy and/or chemotherapy. The standard total mesorectal excision (TME) was performed for rectal cancer patients. After surgery, patients were treated with radiotherapy and/or chemotherapy or not according to body situation, tumor location, and TNM staging system. Patients with rectal cancer were treated with adjuvant chemoradiotherapy (40-50Gy/2Gy/20-25F and Xeloda), while patients with colon cancer were managed with Xeloda plus 5-Fu regimens. A total of 224 (10.9%, 224/2051) patients who were at high risk (venous/lymphatic invasion, poor differentiation, or advanced stage) of local recurrence (LR) and distant metastasis (DM) due to rejection, poor physical condition or side effects, did not receive adjuvant therapy.

### Pathologic examination and staging strategy

Slides from all resected specimens were reexamined by local pathologists who were blinded to the patients' clinical outcomes according to a standardized protocol including determination of the AJCC TNM7 classification, differentiation degree, histological type, numbers of resected and involved lymph nodes, and presence or absence of lymphatic or venous invasion. Negative, microscopic and macroscopic involvement was recorded as R0, R1 and R2, respectively. TDs were assessed using the 3-mm (TNM5) and contour (TNM6) rules [5, 6]. Regular tumor nodules were classified as positive LN. Irregular nodules without any residual tissues of LN were considered as TD if their diameters were > 3mm measured with a ruler. Otherwise, we irregular nodules were considered as pT3 if their diameters were ≤3mm. We counted TDs as pLNs in a new pN category, which included four tiers as follows: npN1a (one tumor node), npN1b (two to three tumor nodes), npN2a (four to six tumor nodes), and npN2b (≥ seven tumor nodes). All patients were classified depending on TNM7 [8]. A new LNR (nLNR) category was defined with four tiers as follows: nLNR1: ≤ 0.25; nLNR2: > 0.25 but ≤ 0.50; nLNR3: > 0.50 but ≤ 0.75; nLNR4: > 0.75.

### Follow-up

Follow-up results were collected from all seven hospitals. The last follow-up date for this study was May 2015. The median time of follow-up was 61 months (range: 2-136 months). All time-to-event end points were measured from date of radical surgery. DFS and OS were calculated from radical surgery to finding evidence of local recurrence and/or distant metastasis, and death of any cause, respectively.

### Statistical analysis

LR and DM analyses were performed for all eligible patients who received R0 resection. Statistical analysis was performed using SPSS software (version 20.0). Differences were evaluated with the log-rank test. Multivariate models were performed using the Cox proportional hazards model. All significant variables in the univariate analysis were included in multivariate Cox regression models in a forward-step procedure. The variables were entered into the regression models with increasing complexity, in order, according to clinical relevance, and significance was assessed using variance analysis. The predictive power of the individual model was evaluated using a receiver operating characteristic (ROC) curve. A two-sided *P* value <0.05 was considered to be of statistical significance.
